# Serum interleukin-6 as a neuroinflammatory biomarker across the spectrum of neurological disorders: a large-scale retrospective cohort study of 6,465 individuals

**DOI:** 10.3389/fimmu.2026.1825206

**Published:** 2026-05-13

**Authors:** Lingjun Zhao, Zhaoming Tang, Chengliang Zhu

**Affiliations:** 1 Department of Clinical Laboratory, Renmin Hospital of Wuhan University, Wuhan, Hubei, China; 2 Department of Clinical Laboratory, Union Hospital, Tongji Medical College, Huazhong University of Science and Technology, Wuhan, Hubei, China

**Keywords:** Biomarkers, emergency triage, interleukin-6, neuro-immunology, neuroinflammation, neurological disorders, traumatic brain injury

## Abstract

**Background:**

Neurological disorders have overlapping clinical manifestations, creating an urgent need for accessible biomarkers to aid differential diagnosis. Interleukin-6 (IL-6) is a core neuroinflammatory mediator, yet its expression profile across the full spectrum of neurological disorders remains poorly characterized in large-scale cohorts using a uniform detection platform. We aimed to map serum IL-6 levels across 10 categories of neurological disorders, with healthy individuals as controls, identify its independent predictors, and evaluate its multi-scenario diagnostic performance.

**Methods:**

We conducted a retrospective observational non-interventional cohort study at Renmin Hospital of Wuhan University (a tertiary academic medical center in Wuhan, China) enrolling 6,465 individuals (515 healthy controls, 5,950 patients across 10 neurological disease categories) who underwent serum IL-6 testing via cytometric bead array (CBA) between January 2018 and September 2025. Statistical analyses included Kruskal–Wallis test, multivariable linear regression, receiver operating characteristic (ROC) curve analysis, and decision curve analysis (DCA). Nested logistic regression models were constructed to evaluate the incremental diagnostic value of IL-6 beyond age and sex.

**Results:**

Serum IL-6 levels showed significant heterogeneity across disease categories (p < 0.001). The highest elevations were observed in traumatic brain injury (TBI), metabolic/toxic encephalopathy, and hemorrhagic stroke (median: 59.93, 28.41, 24.09 pg/mL), with only mild increases in neurodegenerative diseases and intracranial tumors. Disease category, age (U-shaped association), and male sex were independent predictors of IL-6 levels (all p < 0.001). IL-6 achieved good diagnostic performance for distinguishing TBI (AUC 0.974), metabolic/toxic encephalopathy (AUC 0.940), and hemorrhagic stroke (AUC 0.925) from healthy status; notably, IL-6 showed limited discriminatory power for differential diagnosis between TBI and other neurological diseases (AUC = 0.741). Incorporating age and sex significantly improved discrimination for CNS infections (ΔAUC = 0.129, p < 0.0001), with the age+sex-only model achieving an AUC of 0.785 (95% CI 0.75–0.82).

**Conclusion:**

In this large-scale cohort study, we demonstrate that serum IL-6 is a neuroinflammatory biomarker with robust efficacy for screening acute brain injury from healthy status. Its clinical interpretation requires age- and sex-stratified reference intervals, and it holds potential as a preliminary screening indicator for acute neurological conditions in specific clinical settings. The diagnostic utility of IL-6 in unselected emergency triage settings, as well as the proposed clinical interpretation framework, require prospective validation in multi-center, real-world cohorts.

## Highlights

- First large-scale head-to-head mapping of serum IL-6 profiles across 10 neurological disease categories with healthy controls using a single uniform detection platform.
- Identified a quantitative disorder-associated IL-6 gradient between acute and chronic neurological disorders.
- Validated U-shaped age and sex effects as independent predictors of serum IL-6 levels via robust multivariable regression analysis.
- Demonstrated good diagnostic efficacy of IL-6 for screening acute brain injury from healthy status, with limited discriminatory power for inter-disease differential diagnosis.
- Proposed a hypothesis-generating clinical interpretation framework for serum IL-6 testing that requires prospective multi-center validation.

## 1 Introduction

Neurological disorders, including traumatic brain injury (TBI), ischemic and hemorrhagic cerebrovascular diseases, and neurodegenerative diseases (e.g., Alzheimer’s disease, Parkinson’s disease), are leading global causes of disability and mortality (1). Despite having distinct pathophysiological mechanisms, these neurological disorders frequently exhibit overlapping neuroinflammatory signatures across both acute and chronic disease phases, rendering early and accurate differential diagnosis based on clinical features alone extremely challenging (2).

Neuroimaging, including computed tomography (CT) and magnetic resonance imaging (MRI), is currently the clinical gold standard for the diagnosis of most brain disorders. However, its accessibility is severely limited in low-resource settings, and it often fails to detect specific pathological alterations in the ultra-early stages of disease progression (3–5). Therefore, there is an urgent unmet clinical need to develop rapid, cost-effective, and widely accessible humoral biomarkers to assist clinical decision-making and guide individualized precision treatment (6).

Interleukin-6 (IL-6) serves as a central regulator of neuroinflammation in the central nervous system (CNS) (2, 7, 8), operating through two mechanistically distinct pathways: canonical signaling and trans-signaling. Canonical signaling requires membrane-bound IL-6 receptor α (mIL-6Rα), expressed predominantly on hepatocytes and select immune cells, whereas trans-signaling involves the soluble IL-6 receptor (sIL-6R) complexing with IL-6 to activate the ubiquitously expressed co-receptor glycoprotein 130 (gp130), thereby extending IL-6 responsiveness to nearly all cell types in the CNS—including neurons, astrocytes, microglia, and brain endothelial cells (9–12).

In acute neurological insults such as traumatic brain injury (TBI), hemorrhagic/ischemic stroke, and metabolic/toxic encephalopathy, blood–brain barrier (BBB) disruption is the core driver of pathological IL-6 trans-signaling activation: BBB leakage permits peripheral sIL-6R and inflammatory mediators to infiltrate the CNS parenchyma (13, 14), which in turn drives microglial phagocytosis, astrocyte reactivity, CCL2-mediated further BBB breakdown, neuronal hyperexcitability, and a self-sustained cycle of neuroinflammation (8, 15). Elevated serum IL-6 levels have been consistently correlated with worse clinical outcomes in patients with stroke and TBI. Meanwhile, circulating neuroinflammatory biomarkers including IL-6, IL-8, S100β, and neuron-specific enolase (NSE) have been integrated into clinically validated point-of-care panels (e.g., the FDA-cleared i-STAT TBI Plasma Biomarker Panel) for rapid assessment of BBB integrity and injury severity in emergency settings (16, 17).

Accumulating evidence has refined the long-standing notion that canonical signaling mediates predominantly neuroprotective effects in chronic neurodegeneration: IL-6 trans-signaling has now been identified as a critical driver of pathological progression across diverse chronic neurological disorders. In amyotrophic lateral sclerosis (ALS), trans-signaling promotes chronic neuroinflammation and protein aggregate-mediated microglial activation (18); in Alzheimer’s disease models, hippocampal and hypothalamic IL-6 pathway activation correlates with cognitive impairment, and neutralization of IL-6 or inhibition of downstream STAT3 signaling alleviates these deficits. Similarly, in perioperative neurocognitive disorders, trans-signaling (rather than canonical signaling) is the primary mediator of neuronal dysfunction and synaptic remodeling (19, 20).

Therapeutically, selective inhibition of IL-6 trans-signaling preserves the beneficial effects of canonical signaling while mitigating pathological neuroinflammation. Soluble gp130Fc (sgp130Fc; e.g., olamkicept) blocks IL-6/sIL-6R complexes without affecting IL-11 signaling when engineered with enhanced specificity, and demonstrates efficacy in reducing microglial activation, neuronal death, BBB permeability, and long-term functional deficits in TBI, stroke, and ALS models (21). Clinically, the IL-6 receptor monoclonal antibody satralizumab has shown promising efficacy in neuroimmunological disorders, with Phase III trials supporting its utility in conditions involving dysregulated IL-6 signaling.

Given IL-6’s dual role—context-dependent pro-inflammatory trans-signaling versus regulated canonical signaling—and its strong association with BBB dysfunction, neuroinflammation, and validated clinical biomarkers, IL-6 (particularly the sIL-6R/sgp130 ratio) holds significant value as a dynamic diagnostic and prognostic indicator across acute and chronic neurological conditions (20). However, the disease-specific expression profile of serum IL-6 across the full spectrum of neurological disorders, as well as the independent effects of age and sex on circulating IL-6 levels, remain to be systematically characterized in large-scale, uniform-platform cohorts. Targeting trans-signaling also represents a precision strategy to disrupt pathological inflammation while preserving the regenerative functions of IL-6, further highlighting the clinical need for standardized interpretation of circulating IL-6 levels in neurological disease management.

### 1.1 Research gaps and incremental contributions of this study

Existing clinical studies on IL-6 in neurological diseases are predominantly restricted to single disease entities or small subsets of related disorders, with three critical unaddressed gaps hindering its standardized clinical application (22–24):

1. Quantitative gradient data gap: While prior studies have reported elevated IL-6 levels in individual acute brain injury disorders and mild increases in chronic neurodegenerative diseases, no large-scale study has performed a head-to-head comparison of IL-6 expression patterns across the full spectrum of acute and chronic neurological disorders using a single uniform detection platform. The relative magnitude of IL-6 elevation across distinct disease categories, and the quantitative boundary between acute and chronic neurological disorders, remain uncharacterized in a single-cohort setting.
2. Multi-scenario diagnostic evidence gap: Insufficient high-quality evidence exists on the diagnostic efficacy of IL-6 in multi-scenario clinical applications, including disease screening from healthy populations and inter-disease differential diagnosis. Most prior studies have focused on single-disease vs. healthy control comparisons, with no systematic evaluation of IL-6’s discriminatory power across related neurological disorders.
3. Confounding factor validation gap: Although age and sex have been reported to influence circulating IL-6 levels in general populations, no robust statistical validation of their independent effects on IL-6 levels in a large neurological disease cohort has been performed, nor has their incremental value for diagnostic performance optimization been systematically evaluated.

To fill these gaps, we conducted a large-scale retrospective cohort study enrolling 6,465 individuals to systematically characterize serum IL-6 expression profiles across 10 neurological disease categories, identify independent predictors of IL-6 concentrations, and evaluate its diagnostic performance in multi-level clinical scenarios.

## 2 Materials and methods

### 2.1 Ethics statement

This retrospective observational cohort study was conducted in strict accordance with the Declaration of Helsinki. The study protocol was approved by the Institutional Review Board of Renmin Hospital of Wuhan University (Approval No. WDRY2025-K145). The requirement for written informed consent was waived by the IRB, due to the retrospective design of the study and the use of fully anonymized clinical data with no risk to participant privacy. For minor participants under 18 years of age, the requirement for written informed consent from their legal guardians was also waived by the Institutional Review Board, due to the fully anonymized retrospective study design.

Clinical trial number: not applicable. This study is a retrospective observational non-interventional cohort study, which does not meet the WHO/ICMJE definition of a clinical trial, and thus is exempt from clinical trial registration requirements.

### 2.2 Study design and study population

This study consecutively enrolled 6,465 individuals who underwent serum IL-6 testing for clinical indications or routine health examinations at Renmin Hospital of Wuhan University between January 2018 and September 2025.

#### 2.2.1 Inclusion and exclusion criteria

Inclusion criteria:

1. Healthy control group: Individuals who underwent routine physical examinations during the same enrollment period (January 2018 to September 2025), with no history of neurological disease, acute infection within 4 weeks prior to testing, chronic inflammatory/autoimmune diseases (e.g., psoriasis, rheumatoid arthritis, inflammatory bowel disease), use of immunomodulatory medications within 3 months prior to testing, or abnormal findings in cranial imaging.
2. Neurological disease group: Patients admitted with a single primary diagnosis of neurological disorders, confirmed by clinical manifestations, neuroimaging (CT/MRI) findings, and laboratory test results, in accordance with the International Classification of Diseases, 10th Revision (ICD-10) codes.

Exclusion criteria:

1. Patients admitted with any non-neurological primary diagnosis, including systemic infection, autoimmune diseases, malignant tumors, major surgery within 3 months, or end-stage organ failure;
2. Patients with a history of chronic inflammatory/autoimmune diseases or use of immunomodulatory medications within 3 months prior to admission;
3. Patients with incomplete clinical data or invalid IL-6 test results.

#### 2.2.2 Study group classification

The cohort was divided into two groups:

- Healthy control group (n = 515): Frequency-matched to the overall neurological disease group by age and sex distribution to minimize demographic confounding. All participants were tested using the same CBA platform and reagent batches as the neurological disease group, with consistent laboratory testing protocols.
- Neurological disease group (n = 5,950): Classified into 10 disease categories based on ICD-10 codes: ischemic cerebrovascular disease (n = 2,077), hemorrhagic cerebrovascular disease (n = 1,782), traumatic brain injury (TBI, n = 386), CNS infection/inflammation (n = 375), neurodegenerative diseases (n = 196), metabolic/toxic encephalopathy (n = 168), intracranial tumors (n = 158), hydrocephalus/CSF disorders (n = 78), other cerebrovascular diseases (n = 712), and other neurological disorders (n = 18).
- The “other cerebrovascular diseases” and “other neurological disorders” groups were excluded from all primary inferential statistical analyses (including regression modeling and diagnostic performance evaluation) due to ambiguous classification and small sample size (n=18 for the latter group), with only descriptive statistics presented to ensure analytical rigor.
- The final analytical sample size for regression modeling was 5,735 individuals.

Sample size estimation was performed based on the primary outcome of IL-6 diagnostic efficacy for TBI. Referring to previous studies reporting an AUC of 0.95 for serum IL-6 in distinguishing TBI from healthy controls, with a two-sided α of 0.05 and a statistical power of 90%, the minimum required sample size was 128 cases and 128 controls. The final enrolled sample size (386 TBI cases and 515 healthy controls) far exceeded the estimated minimum, ensuring sufficient statistical power for all analyses. Sample size estimation was performed using the pROC package in R software (version 4.5.0).

### 2.3 Data collection and IL-6 measurement

All clinical and laboratory data were extracted from the hospital’s standardized electronic health record system, including demographic information (age, sex), definitive clinical diagnosis, and serum IL-6 concentration test results.

For healthy controls: Venous blood samples were collected in the morning after an overnight fast.

For neurological disease patients: Venous blood samples were collected within 24 hours of hospital admission, before any surgical intervention or immunomodulatory treatment. For all traumatic brain injury patients, venous blood samples were collected immediately upon hospital admission, before any surgical intervention or medical treatment.

Serum IL-6 concentrations were measured using the cytometric bead array (CBA) method with a commercial human IL-6 detection kit (Cat. No. CBA-IL6-001, Zhongshan Bio-Tech Co., Ltd, Suzhou, China) on a BD FACSCanto II flow cytometer (BD Biosciences, San Jose, CA, USA). The assay was performed strictly following the manufacturer’s instructions, with intra-assay coefficient of variation (CV) < 5% and inter-assay CV < 8%. The linear detection range was 1.5–5000 pg/mL, with a lower limit of quantification (LLOQ) of 1.5 pg/mL. Values below the LLOQ were recorded as 1.5 pg/mL for statistical analysis, in accordance with international standard laboratory practice.

### 2.4 Statistical analysis

All statistical analyses were performed using R software (version 4.5.0; R Foundation for Statistical Computing, Vienna, Austria). A two-sided p-value < 0.05 was considered statistically significant for all analyses, unless otherwise specified.

Normality test and data transformation: Continuous variables including serum IL-6 concentrations showed a severely skewed distribution (Shapiro-Wilk test, p < 0.001). Natural logarithmic transformation (ln[IL-6 + 1]) was applied to normalize the distribution, designated as log_IL6.

Intergroup comparisons: The Kruskal–Wallis test was used to assess overall differences among the study groups. *Post-hoc* pairwise comparisons were conducted using Dunn’s test, with the Benjamini–Hochberg false discovery rate (FDR) correction applied for multiple comparisons (FDR q-value < 0.05 considered significant). Effect sizes were quantified by reporting ϵ² for each comparison.

Independent predictor analysis: Multivariable linear regression model was constructed with log_IL6 as the dependent variable, and disease category, age (linear and quadratic terms), and sex as independent variables. Heteroskedasticity-consistent standard errors (HC3 estimator) were used, with model robustness validated via Breusch–Pagan test, median regression, and Cook’s distance sensitivity analysis.

Diagnostic performance evaluation: Receiver operating characteristic (ROC) curve analysis was performed to calculate AUC, optimal cutoff value (maximizing Youden’s index), sensitivity, specificity, positive/negative likelihood ratio, and positive/negative predictive value. 95% confidence intervals (CIs) were calculated and reported for all optimal cutoff values via bootstrap resampling (1000 iterations). DeLong test was used to compare AUC differences between models, with bootstrap resampling (1000 iterations) for internal validation. Decision curve analysis (DCA) was performed to evaluate the net clinical benefit of the models.

To evaluate the incremental diagnostic value of IL-6 beyond age and sex for CNS infections, four nested logistic regression models were constructed:

1. Null model (intercept only).
2. Demographics model (age + sex).
3. Univariate model (log_IL6 only).
4. Full model (age + sex + log_IL6).

Sensitivity analysis using propensity score matching (1:1 nearest neighbor matching, caliper = 0.2 SD) was performed to adjust for age differences between the CNS infection group and healthy controls, and the diagnostic performance of IL-6 was re-evaluated in the matched cohort.

Planned supplementary sensitivity analysis: We originally planned to perform a sensitivity analysis by further excluding patients with concurrent in-hospital systemic infection, extracranial polytrauma, or sepsis-associated encephalopathy (which were not fully excluded by the baseline enrollment criteria) to validate the robustness of the disease-specific IL-6 gradient. However, due to the retrospective design of the study, complete comorbidity data for these conditions were not systematically collected for all participants, and this analysis is reserved for future prospective studies.

## 3 Results

### 3.1 Baseline characteristics of the study cohort

A total of 6,465 participants were included in the final study cohort, and their demographic and clinical characteristics are summarized in **Table 1**. The healthy control group had a mean age of 51.4 ± 14.7 years, with 58.6% male participants. Significant differences in age and sex distribution were observed across the disease categories: the CNS infection/inflammation group had the youngest mean age (27.7 ± 22.4 years), while the neurodegenerative disease group had the oldest mean age (64.6 ± 13.7 years). With the exception of the intracranial tumor group (48.1% male), all other disease groups had a male proportion exceeding 52.6%, with the highest proportion observed in the TBI group (74.4%).

**Table 1:** Demographic and clinical characteristics of the study cohort stratified by disease category.

Disease category	n	Male, n (%)	Age, years (mean ± SD)	IL-6, pg/mL (median [IQR])	log_IL6 (mean ± SD)
Healthy controls	515	302 (58.6)	51.4 ± 14.7	2.02 (1.50–3.56)	1.33 ± 0.64
Ischemic cerebrovascular disease	2077	1508 (72.6)	62.5 ± 13.4	14.00 (4.78–38.81)	2.90 ± 1.47
Hemorrhagic cerebrovascular disease	1782	1214 (68.1)	55.8 ± 12.9	24.09 (9.74–72.17)	3.44 ± 1.53
Traumatic brain injury (TBI)	386	287 (74.4)	50.8 ± 18.4	59.93 (20.67–137.47)	4.16 ± 1.46
CNS infection/ inflammation	375	217 (57.9)	27.7 ± 22.4	8.34 (2.88–27.21)	2.52 ± 1.45
Neurodegenerative diseases	196	103 (52.6)	64.6 ± 13.7	5.04 (1.89–17.83)	2.25 ± 1.42
Metabolic/toxic encephalopathy	168	126 (75.0)	55.7 ± 17.4	28.41 (10.12–113.03)	3.80 ± 1.78
Intracranial tumors	158	76 (48.1)	48.9 ± 18.6	5.38 (2.50–21.83)	2.34 ± 1.43
Hydrocephalus/CSF disorders	78	42 (53.8)	43.1 ± 24.1	12.54 (5.66–40.90)	2.94 ± 1.37
Other cerebrovascular diseases	712	472 (66.3)	62.2 ± 15.1	14.64 (5.25–40.29)	2.92 ± 1.44
Other neurological disorders*	18	10 (55.6)	41.2 ± 18.8	4.77 (2.01–10.80)	2.11 ± 1.42

CNS, central nervous system; CSF, cerebrospinal fluid; IQR, interquartile range; SD, standard deviation.

* This group was excluded from all primary inferential statistical analyses due to small sample size and ambiguous classification.

### 3.2 Heterogeneity of serum IL-6 levels across neurological disease categories

**Figure 1:**
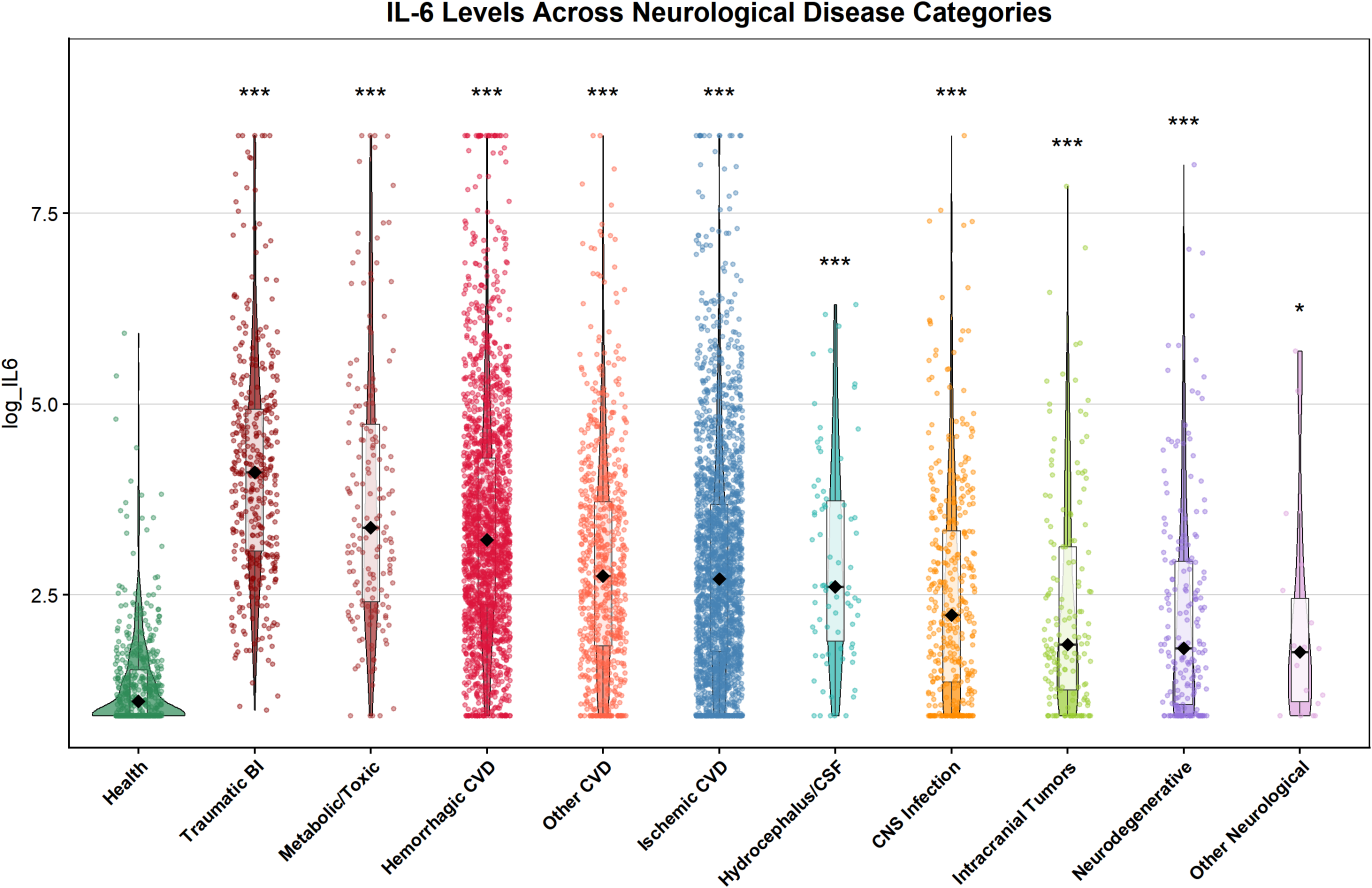
Distribution of log-transformed serum IL-6 levels across neurological disease categories.

Serum IL-6 levels exhibited significant heterogeneity across the 11 study groups (10 neurological disease categories + 1 healthy control group) (Kruskal-Wallis H = 1333.37, df = 10, p < 0.001, ϵ² = 0.205). Compared with healthy controls, serum IL-6 levels were significantly elevated in all disease categories (FDR-corrected p < 0.05), with a distinct disease-specific gradient elevation pattern (**Figure 1**).

The highest IL-6 elevations were observed in acute brain injury and encephalopathy: the median IL-6 level in the TBI group was 59.93 pg/mL (~30-fold higher than healthy controls [2.02 pg/mL]), followed by the metabolic/toxic encephalopathy group (28.41 pg/mL) and hemorrhagic cerebrovascular disease group (24.09 pg/mL). In contrast, only mild elevations were detected in chronic neurological conditions, including neurodegenerative diseases (5.04 pg/mL) and intracranial tumors (5.38 pg/mL).

Boxes represent the median and interquartile range; whiskers extend to 1.5 × the interquartile range. Outliers are plotted as individual data points. *p < 0.001 versus healthy controls (Dunn’s test with FDR correction). Abbreviations: CNS, central nervous system; CSF, cerebrospinal fluid; CVD, cerebrovascular disease; TBI, traumatic brain injury.

### 3.3 Independent predictors of serum IL-6 concentrations

Multivariable linear regression analysis (**Table 2**) further quantified the independent contributions of disease category, age, and sex to serum IL-6 levels. The overall regression model was statistically significant (F_11_,_5723_ = 138.01, p < 0.001, adjusted R² = 0.208), with all analyses performed after excluding the ambiguous “other” disease groups (n=5,735).

**Table 2:** Multivariable linear regression analysis of factors associated with log-transformed IL-6 levels.

Variable	β	SE_robust	p_robust	95% CI
Intercept	1.2404	0.1333	<0.001	(0.9790, 1.5017)
Age (linear term)	-0.0059	0.0045	0.1877	(-0.0147, 0.0029)
Age² (quadratic term)	0.0002	0.00005	<0.001	(0.0001, 0.0003)
Sex (Female vs. Male)	-0.3469	0.0406	<0.001	(-0.4265, -0.2673)
Traumatic brain injury	2.7601	0.0959	<0.001	(2.5721, 2.9480)
CNS infection/inflammation	1.3518	0.106	<0.001	(1.1440, 1.5595)
Ischemic cerebrovascular disease	1.3449	0.0718	<0.001	(1.2042, 1.4856)
Hemorrhagic cerebrovascular disease	2.0183	0.0713	<0.001	(1.8784, 2.1581)
Intracranial tumors	1.0529	0.1292	<0.001	(0.7995, 1.3062)
Neurodegenerative diseases	0.7258	0.1205	<0.001	(0.4895, 0.9621)
Metabolic/toxic encephalopathy	2.3249	0.1265	<0.001	(2.0769, 2.5729)
Hydrocephalus/CSF disorders	1.6562	0.1735	<0.001	(1.3160, 1.9964)

CI, confidence interval; CNS, central nervous system; SE, standard error.

The overall regression model included 12 independent variables (intercept + 11 predictive variables), resulting in a residual degree of freedom of 5723 (5735-12).

**Figure 2:**
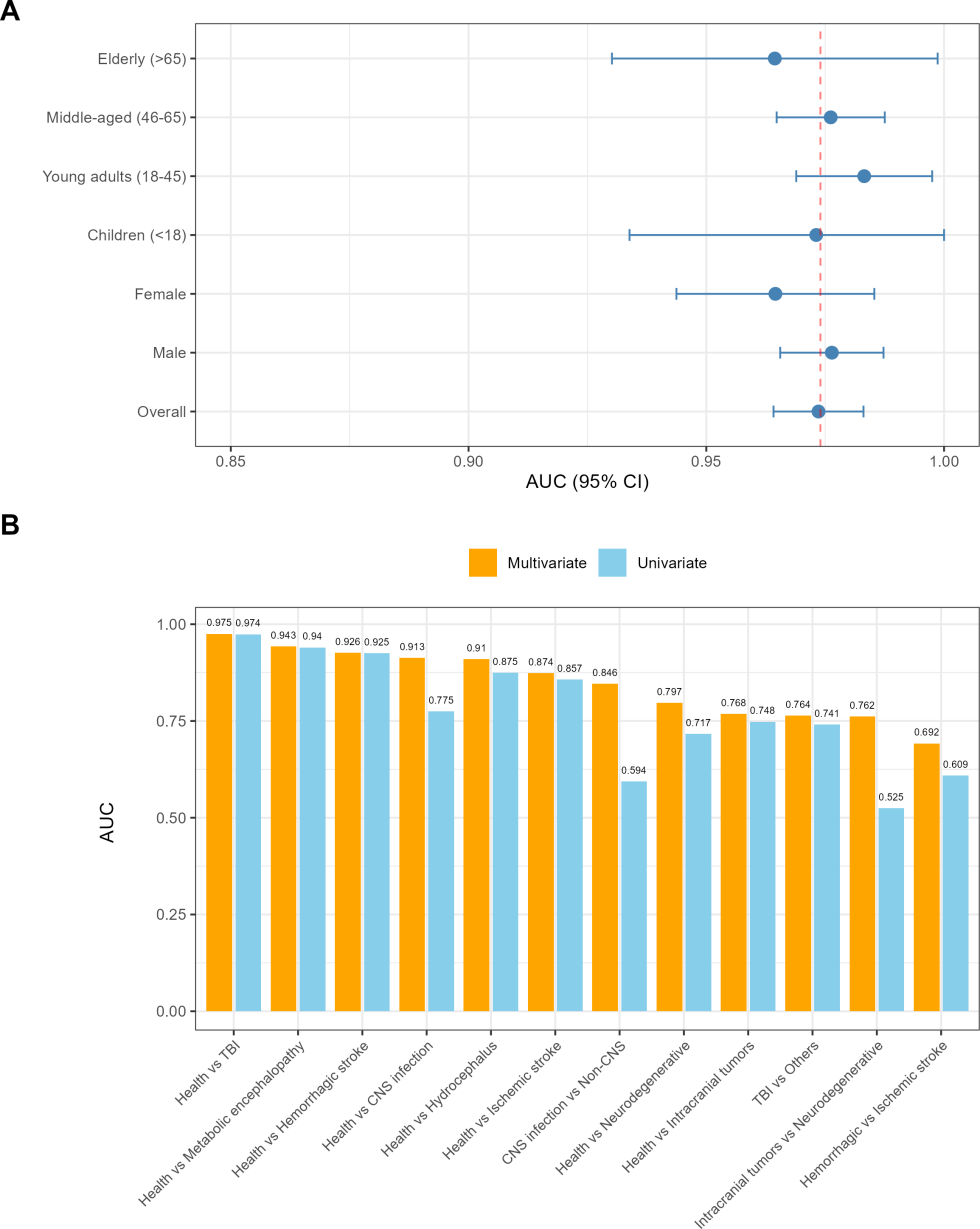
Multivariable linear regression analysis of factors associated with log-transformed serum interleukin-6 (log_IL6) levels. **(A)** Forest plot of disease-specific effects relative to healthy controls. Points represent β coefficients from the multivariable linear regression model (adjusted for age, age squared term, sex, and disease category). Error bars indicate 95% confidence intervals (CI) calculated using heteroscedasticity-consistent standard errors (HC3 estimator). All listed disease categories showed significantly higher log_IL6 levels compared with the healthy control reference group (all P < 0.001). TBI, traumatic brain injury; MET, metabolic/toxic encephalopathy; HEM, hemorrhagic cerebrovascular disease; HYD, hydrocephalus/cerebrospinal fluid disorders; ISCH, ischemic cerebrovascular disease; CNS, central nervous system infection/inflammation; TUMOR, intracranial tumors; ND, neurodegenerative diseases. **(B)** Age and sex effects on predicted log_IL6 levels in the healthy control subgroup. Solid lines represent predicted log_IL6 values derived from the multivariable model, with disease category fixed at the healthy control group. Shaded ribbons indicate 95% confidence bands for the predicted values. A significant U-shaped association between age and log_IL6 was observed, with higher levels in children and elderly individuals and lower levels in middle-aged adults. Male participants exhibited consistently higher predicted log_IL6 levels than Female participants across all age ranges. **(C)** Age trajectories of predicted log_IL6 levels in female patients stratified by disease category. Curves show predicted log_IL6 values as a function of age for five representative disease categories in female patients, derived from the same multivariable linear regression model. The U-shaped age pattern observed in healthy controls was preserved across all disease categories, and the relative ranking of disease-specific effects remained consistent with the forest plot in panel **(A)** Shaded ribbons represent 95% confidence intervals for the predicted values. **(D)** Age trajectories of predicted log_IL6 levels in male patients stratified by disease category. Curves illustrate predicted log_IL6 values across age for the same five representative disease categories in male patients, consistent with the analysis in panel **(C)** Compared with female patients, male patients had higher predicted log_IL6 levels for each disease group, while maintaining the same U-shaped age association and relative ranking of disease-specific effects. All analyses for this figure were performed after excluding the “other cerebrovascular diseases” and “other neurological disorders” groups, with a final analytical sample size of n = 5,735, and healthy controls as the reference group.

Disease category was the strongest independent predictor of IL-6 levels, with effect sizes (β) decreasing in the following order: TBI (β = 2.760), metabolic/toxic encephalopathy (β = 2.325), hemorrhagic cerebrovascular disease (β = 2.018), hydrocephalus/CSF disorders (β = 1.656), CNS infection/inflammation (β = 1.352), ischemic cerebrovascular disease (β = 1.345), intracranial tumors (β = 1.053), and neurodegenerative diseases (β = 0.726) (**Figure 2A**). All disease categories were associated with significantly higher log-IL6 levels compared with healthy controls (all p < 0.001).

Age exhibited a significant U-shaped association with log_IL6 (quadratic term p < 0.001), with higher IL-6 levels observed in childhood and old age, and lower levels in middle age. A significant sex effect was also identified, with female participants having a 0.347 lower mean log_IL6 value than male participants (95% CI -0.424 to -0.270, p < 0.001). This age- and sex-dependent pattern was consistent in the healthy control subgroup (**Figure 2B**), and was preserved across all neurological disease categories in both female (**Figure 2C**) and male patients (**Figure 2D**). Notably, the relative ranking of disease-specific IL-6 elevation remained stable across all age groups and both sexes, further confirming the disease-specific gradient of serum IL-6 levels.

Detailed serum IL-6 level comparisons across all neurological diseases stratified by sex and age subgroups are presented in **Supplementary Table 1**.

### 3.4 Diagnostic performance of serum IL-6

#### 3.4.1 Diagnostic performance for distinguishing healthy status from neurological disease

Serum IL-6 exhibited moderate to good diagnostic performance for differentiating healthy controls from patients with various neurological diseases (**Table 3**). For TBI, IL-6 achieved an AUC of 0.974 (95% CI 0.964–0.983). At the optimal cutoff value of 10.25 pg/mL (95% CI 6.75–13.53), the sensitivity was 0.915, specificity was 0.938, positive likelihood ratio (PLR) was 14.72, and negative likelihood ratio (NLR) was 0.091. Good diagnostic performance was also observed for metabolic/toxic encephalopathy (AUC = 0.940, 95% CI 0.917–0.962) and hemorrhagic cerebrovascular disease (AUC = 0.925, 95% CI 0.912–0.938). Even for neurodegenerative diseases, which had the lowest discriminatory power, the AUC remained 0.717 (95% CI 0.671–0.762), which was significantly higher than the random guess threshold of 0.5 (p < 0.001), supporting its potential auxiliary screening value for chronic neurological conditions.

**Table 3:** Diagnostic performance of serum IL-6 for differentiating healthy controls from individual disease groups.

Comparison	AUC (95% CI)	Cutoff (pg/mL) (95% CI)	Sensitivity	Specificity	PPV	NPV	Sample size (case/control)
TBI	0.974 (0.964-0.983)	10.25 (6.75–13.53)	0.915	0.938	0.917	0.936	386/515
CNS infection	0.775 (0.743-0.808)	5.99 (4.18–8.17)	0.624	0.852	0.755	0.757	375/515
Ischemic stroke	0.857 (0.840-0.873)	5.92 (4.64–7.46)	0.745	0.850	0.953	0.452	2077/515
Hemorrhagic stroke	0.925 (0.912-0.938)	6.44 (5.92–7.88)	0.868	0.868	0.958	0.654	1782/515
Intracranial tumors	0.748 (0.701-0.795)	4.29 (4.28–5.95)	0.690	0.736	0.445	0.886	158/515
Neurodegenerative	0.717 (0.671-0.762)	4.46 (4.12–9.60)	0.622	0.746	0.482	0.838	196/515
Metabolic encephalopathy	0.940 (0.917-0.962)	7.56 (5.95–8.80)	0.887	0.897	0.738	0.960	168/515
Hydrocephalus	0.875 (0.828-0.923)	4.92 (4.87–8.21)	0.872	0.775	0.370	0.976	78/515

AUC, area under the receiver operating characteristic curve; CI, confidence interval; CNS, central nervous system; NPV, negative predictive value; PPV, positive predictive value; TBI, traumatic brain injury.

#### 3.4.2 Diagnostic performance for differential diagnosis between disease categories

The discriminatory power of IL-6 alone was markedly reduced when used for differential diagnosis between specific disease pairs (**Table 4**). The AUC for differentiating TBI from all other neurological diseases was 0.741 (95% CI 0.719–0.762), with a specificity of only 0.541 at the optimal cutoff, indicating limited clinical utility for this differentiation. The AUC for distinguishing hemorrhagic stroke from ischemic stroke was 0.609 (95% CI 0.592–0.627), while the AUC for differentiating intracranial tumors from neurodegenerative diseases was only 0.525 (95% CI 0.465–0.585), which was only marginally higher than random guessing. The full diagnostic performance of serum IL-6 for pairwise differential diagnosis between all neurological disease categories is provided in **Supplementary Table 2**.

**Table 4:** Diagnostic performance of serum IL-6 for differential diagnosis between key disease pairs.

Comparison	AUC (95% CI)	Cutoff (pg/mL) (95% CI)	Sensitivity	Specificity	PPV	NPV	Sample size (case/control)
TBI vs. Others	0.741 (0.719–0.762)	17.22 (13.14–46.28)	0.826	0.541	0.115	0.977	386/5349
CNS infection vs. Non-CNS	0.594 (0.564–0.623)	18.60 (4.00–22.67)	0.675	0.475	0.046	0.918	375/5360
Hemorrhagic vs. Ischemic stroke	0.609 (0.592–0.627)	15.19 (7.48–18.39)	0.664	0.503	0.534	0.636	1782/2077
Intracranial tumors vs. Neurodegenerative	0.525 (0.465–0.585)	2.88 (2.76–43.73)	0.823	0.250	0.469	0.636	158/196

AUC, area under the receiver operating characteristic curve; CI, confidence interval; CNS, central nervous system; NPV, negative predictive value; PPV, positive predictive value; TBI, traumatic brain injury.

#### 3.4.3 Improvement of diagnostic performance by incorporating age and sex

**Figure 3:**
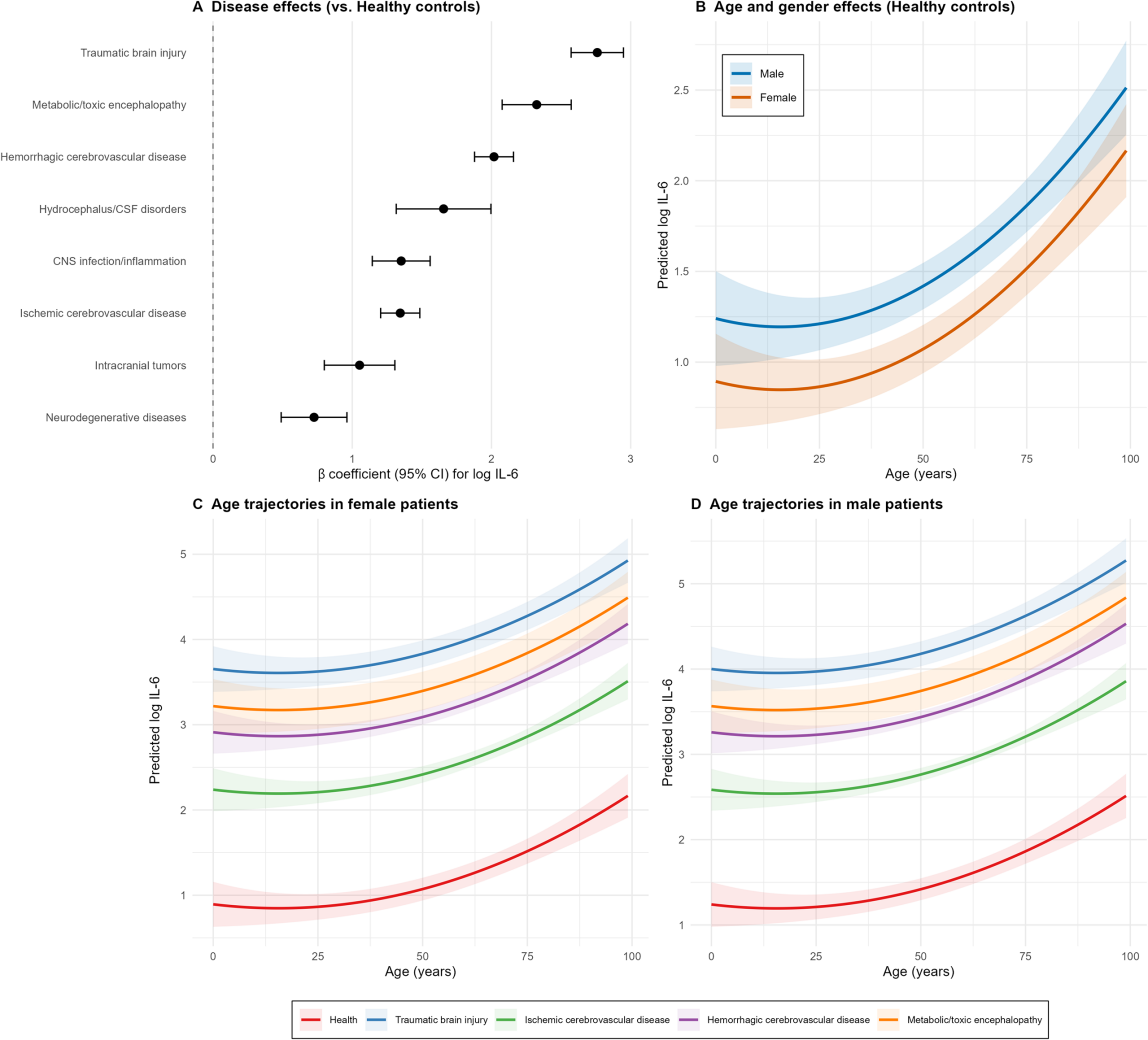
Subgroup diagnostic performance of serum IL-6 and incremental value of age and sex incorporation. **(A)** Forest plot of subgroup-specific AUC values for differentiating TBI from healthy controls. The dashed line indicates the overall AUC of 0.974 for the full TBI cohort. Error bars represent 95% confidence intervals of the AUC values. **(B)** Bar plot comparing bootstrap-corrected AUC values of the univariate model (IL-6 alone) and the multivariable model (IL-6 + age + sex) across all key diagnostic comparison pairs. Values above the bars represent the mean AUC of each model.

Subgroup analysis revealed that the diagnostic performance and optimal cutoff values of IL-6 exhibited significant age and sex dependence. For the differentiation of TBI from healthy controls, the optimal cutoff values were 12.79 pg/mL for males and 6.28 pg/mL for females, and the optimal cutoff for the elderly group (>65 years, 11.20 pg/mL) was higher than that for the pediatric group (<18 years, 5.91 pg/mL), with a significant difference in diagnostic AUC between the two subgroups (p < 0.001, DeLong test) (**Figure 3A**).

The multivariable logistic regression model incorporating log_IL6, age, and sex showed varying degrees of improvement in diagnostic performance compared with the univariate IL-6-only model. For CNS infections, a highly heterogeneous disease group, the AUC increased from 0.775 in the univariate model to 0.914 in the multivariable model (ΔAUC = 0.129, p < 0.0001, DeLong test), with the age+sex-only model achieving an AUC of 0.785 (95% CI 0.75–0.82), confirming the significant incremental diagnostic value of IL-6 beyond demographic factors (**Table 5**). Sensitivity analysis using propensity score matching to adjust for age differences between the CNS infection group and healthy controls further validated the robustness of these findings, with IL-6 achieving an AUC of 0.786 (95% CI 0.75–0.82) in the age-matched cohort. In contrast, for conditions where IL-6 alone already exhibited good diagnostic performance, such as TBI and hemorrhagic stroke, the incremental gain from adding age and sex was negligible (ΔAUC ≤ 0.002, p > 0.05, DeLong test), highlighting the strength of IL-6 as a standalone biomarker for these acute conditions (**Figure 3B**).

**Table 5:** Nested logistic regression models for CNS infection diagnosis.

Model	AUC	95% CI	ΔAUC vs. Demographics	p-value
Null (Intercept)	0.500	0.50–0.50	–	–
Demographics (Age + Sex)	0.785	0.75–0.82	–	< 0.0001 vs. Null
Univariate (log_IL6)	0.775	0.74–0.81	–	< 0.0001 vs. Null
Full Model (Age + Sex + log_IL6)	0.914	0.89–0.93	0.129	< 0.0001 vs. Demographics

AUC, area under the receiver operating characteristic curve; CI, confidence interval; CNS, central nervous system. ΔAUC was calculated as the difference in AUC between the full model and the demographics-only model.

**Figure 4:**
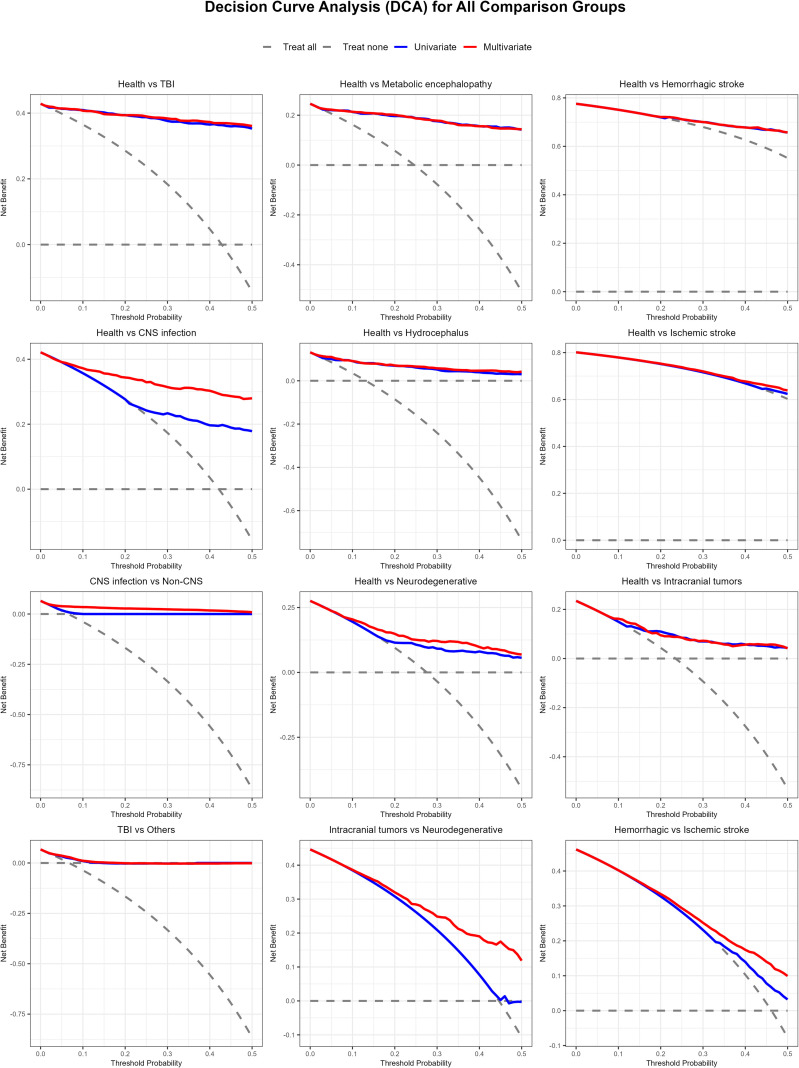
Decision curve analysis of IL-6 in different diagnostic comparisons. Solid lines represent the multivariable model (IL-6 + age + sex), dashed lines represent the univariate IL-6-only model, and gray lines represent the “treat all” and “treat none” reference strategies. Panels show the net clinical benefit of each model across clinically relevant threshold probabilities (0–0.5) for all 12 diagnostic comparison scenarios, including screening from healthy controls and differential diagnosis between neurological disease categories.

Decision curve analysis was performed across all 12 diagnostic comparison scenarios to further validate the clinical utility of the models, with the results presented in **Figure 4**. Across the clinically relevant threshold probability range (0.05–0.3), the multivariable model incorporating IL-6, age and sex consistently provided a higher net clinical benefit than the univariate IL-6-only model, the “treat all” strategy, and the “treat none” strategy for the majority of diagnostic scenarios, particularly for CNS infection screening. For acute brain injury comparisons including TBI, hemorrhagic stroke and metabolic/toxic encephalopathy, the univariate IL-6 model already showed favorable net clinical benefit across most clinically relevant threshold ranges, consistent with the ROC analysis results. A detailed subgroup analysis of IL-6 diagnostic performance for inter-disease differential diagnosis is shown in **Supplementary Table 3**.

## 4 Discussion

To the best of our knowledge, this study is one of the largest single-center systematic evaluations of serum IL-6 expression profiles across the full spectrum of neurological disorders, using a single uniform detection platform. Unlike previous studies restricted to single disease entities or small disorder subsets, this study established a quantitative disease-specific gradient expression profile of serum IL-6 across 10 neurological disease categories, and clearly defined the expression boundary between acute brain injury and chronic neurological diseases, providing robust evidence for the clinical application of IL-6 as a neuroinflammatory biomarker (25).

Our core findings can be summarized into three key points:

1. Serum IL-6 levels exhibit a distinct disease-specific gradient across brain disorders, with significant elevations in conditions characterized by acute neuroinflammation (e.g., TBI, metabolic/toxic encephalopathy, hemorrhagic stroke) and only mild increases in chronic, low-grade inflammatory conditions (e.g., neurodegenerative diseases, intracranial tumors);
2. Age (U-shaped association) and sex (higher levels in males) are two independent and biologically significant modulators of circulating IL-6 levels (22, 23, 26);
3. As a standalone biomarker, IL-6 exhibits good diagnostic efficacy for screening neurological diseases from healthy status, but has limited value for fine-grained differential diagnosis between related disease categories.

The clinical interpretation of IL-6 levels therefore requires stratification based on demographic characteristics.

Our findings are highly consistent with the established pathophysiological roles of IL-6 in neuroinflammation. In the setting of TBI, damaged neurons, along with activated microglia and astrocytes, rapidly release large quantities of IL-6. Concurrently, blood-brain barrier (BBB) disruption facilitates the influx of peripheral inflammatory mediators into the CNS parenchyma, further amplifying neuroinflammation and triggering a robust cytokine storm (27). This BBB disruption-mediated central-peripheral inflammatory coupling mechanism directly explains the high median IL-6 level of nearly 60 pg/mL observed in the TBI group in our study (8, 13, 28).

Similarly, metabolic/toxic encephalopathies (e.g., hepatic encephalopathy, sepsis-associated encephalopathy) are fundamentally driven by systemic inflammatory dysregulation, with the intensity of CNS involvement sufficient to elicit a robust peripheral IL-6 response (29). In contrast, the mild elevation of IL-6 in neurodegenerative diseases reflects their core pathophysiological feature of chronic neuroinflammation, which is characterized by low-grade, sustained microglial activation rather than the acute robust inflammatory response seen in acute brain injury (14, 30).

Notably, despite the neurodegenerative disease group having the highest mean age (64.6 ± 13.7 years), which would theoretically place them in a state of “inflammaging” with elevated baseline inflammatory markers (22), their peripheral IL-6 levels (median 5.04 pg/mL) showed only a mild elevation compared with healthy controls, and remained far lower than those in the acute brain injury groups.

This apparent contradiction can be explained by the central-peripheral compartmentalization hypothesis of neuroinflammation: in chronic neurodegenerative diseases, the inflammatory process is primarily confined within the CNS, while the relatively intact BBB limits the translocation of central inflammatory mediators into the peripheral circulation, resulting in only minimal elevations in serum IL-6 levels (22, 23, 31). Previous studies have demonstrated that CSF IL-6 levels in patients with Alzheimer’s disease can be 5–10 times higher than paired serum levels, and CSF IL-6 shows a significantly stronger correlation with brain atrophy and cognitive decline than peripheral IL-6 (23, 32).

Our findings align perfectly with this pathophysiological model: neurodegenerative diseases exhibit a dissociated pattern of “central high inflammation, peripheral low inflammation”, whereas acute brain injury results in “central-peripheral inflammatory coupling” due to BBB disruption, leading to significant elevations in circulating IL-6 levels. This distinction has critical clinical implications: elevated peripheral IL-6 is a strong indicator of compromised BBB integrity and acute/subacute neuropathological processes; conversely, normal peripheral IL-6 levels in patients with clinically suspected neurodegenerative disease do not rule out CNS inflammatory involvement, and CSF inflammatory marker testing should be considered when clinically indicated.

One of the most clinically translatable findings of our study is the quantitative confirmation of the independent effects of age and sex on serum IL-6 levels. This is the first study to statistically validate a U-shaped association between circulating IL-6 and age using robust heteroskedasticity-consistent regression methods in a large-scale clinical cohort of 6,465 individuals with neurological disorders.

This phenomenon is driven by the developmental characteristics of the immune system: in childhood, the immature immune system is more susceptible to infectious stimuli, leading to stronger inflammatory responses; in advanced age, the body enters a state of inflammaging, with chronically elevated baseline inflammatory tone (33). Similarly, we observed significantly lower IL-6 levels in female participants compared with males, which is consistent with the well-documented anti-inflammatory effects of estrogen and the recognized sex dimorphism in innate and adaptive immune responses (26, 34).

These findings have direct and immediate implications for clinical practice. For example, applying a fixed IL-6 cutoff value (e.g., 10 pg/mL) for TBI screening would inevitably lead to a high rate of false negatives in female and pediatric patients, while potentially generating excessive false positives in elderly male patients. Our study therefore provides compelling evidence for the urgent need to establish age- and sex-stratified reference intervals for the clinical interpretation of serum IL-6 levels, which is a critical unmet need in current laboratory practice.

### 4.1 Clinical positioning of IL-6 relative to established neurobiomarkers

The most clinically relevant finding of this study is the good diagnostic performance of IL-6 for TBI, hemorrhagic stroke, and metabolic/toxic encephalopathy when compared with healthy controls. It is critical to clarify that the only FDA-cleared blood biomarkers for the clinical assessment of mild TBI are glial fibrillary acidic protein (GFAP) and ubiquitin C-terminal hydrolase L1 (UCH-L1) (35), rather than S100β. While S100β is widely used to rule out intracranial lesions in clinical practice, its brain tissue specificity is relatively low (36). GFAP is exclusively expressed in astrocytes, conferring it with extremely high brain tissue specificity (37), whereas IL-6 is a pan-inflammatory cytokine, with elevations observed in a wide range of non-neurological pathological conditions including systemic infection, major surgery, and myocardial infarction (13), resulting in relatively limited disease specificity for neurological disorders (38).

However, IL-6 has unique potential advantages in terms of detection standardization and cost-effectiveness, which support its clinical value as a preliminary screening tool. Mainstream point-of-care testing (POCT) platforms, such as the electrochemistry vertical flow immunodiagnostic (eVFID) device, can achieve ultrasensitive simultaneous detection of IL-6 and procalcitonin (PCT) within 5 minutes, with a limit of quantification as low as 0.1 pg/mL for IL-6 (39). Integrated microfluidic devices also enable simultaneous detection of IL-6 and multiple other biomarkers in saliva within 15 minutes (40), making it a potential auxiliary screening tool in resource-limited settings where neuroimaging or rapid GFAP testing is unavailable. In addition, IL-6 has a longer circulating half-life compared with GFAP, which peaks within hours after TBI (41), reducing its sensitivity to the timing of sample collection after injury and widening the clinical diagnostic window.

Notably, the integrity of the blood-brain barrier (BBB) significantly modulates the correlation between peripheral and central IL-6 levels, which further explains the disease-specific gradient observed in our cohort. In patients with mild cognitive impairment and impaired BBB integrity, serum IL-6 levels showed a significant positive correlation with paired cerebrospinal fluid (CSF) IL-6 levels (r=0.296, P = 0.035), while this correlation was absent in patients with intact BBB (42). A similar pattern has been reported for GFAP, with enhanced serum-CSF correlation in the context of BBB disruption (r=0.652, P<0.001) (42). This mechanistic evidence supports our core finding that elevated peripheral IL-6 levels may indirectly reflect central neuroinflammatory status in the setting of BBB dysfunction, which is the core pathophysiological feature of acute brain injury including TBI and hemorrhagic stroke. This also explains the minimal elevation of serum IL-6 in chronic neurodegenerative diseases, where the BBB remains relatively intact and neuroinflammation is largely confined to the CNS parenchyma.

We therefore propose that the optimal clinical application of IL-6 is not to replace GFAP or UCH-L1, but to serve as a complementary stratification tool in two core clinical scenarios: first, as a preliminary risk assessment tool in resource-limited settings where immediate neuroimaging or rapid GFAP testing is unavailable; second, in combination with GFAP to construct a dual-dimensional assessment model integrating “structural brain injury + systemic inflammatory burden”. Specifically, IL-6 reflects the global inflammatory burden—a concept supported by recent evidence identifying systemic inflammatory cytokines, including IL-6, as key components of the neuroinflammatory response following mild TBI (27, 43)—while GFAP indicates the extent of structural brain injury. Their combination is thus hypothesized to synergistically enhance the prediction of secondary brain injury and functional outcomes.

Recent studies have validated the feasibility of non-invasive simultaneous monitoring of GFAP and IL-6 in sweat using the SWEATSENSER sensor, with limits of quantification of 14 pg/mL and 10 pg/mL respectively (44), providing technical support for the dynamic assessment of neuroinflammatory response after TBI. The large-scale TRACK-TBI cohort study further confirmed that the combination of admission plasma GFAP and UCH-L1 achieved excellent predictive performance for 6-month mortality and unfavorable functional outcomes (Glasgow Outcome Scale-Extended, GOSE ≤4), with an AUC of 0.86–0.89 (41), highlighting the clinical potential of multi-biomarker integrated strategies. Based on this, we propose a testable “dual IL-6-GFAP biomarker strategy” hypothesis: in the emergency assessment of TBI, patients with GFAP >0.05 μg/L (the FDA-cleared clinical cutoff for mild TBI) and IL-6 >20 pg/mL (the high-risk acute brain injury threshold defined in our study cohort) represent a high-risk subtype characterized by “structural brain injury + excessive inflammatory burden,” who may have a significantly higher risk of intracranial hypertension, secondary brain injury, and poor functional prognosis compared with patients with only a single positive biomarker. This hypothesis requires validation in future prospective cohort studies, but provides a theoretical framework for the precision application of IL-6 in TBI management.

While combinatorial approaches utilizing structural and inflammatory biomarkers have been conceptually proposed in recent literature to address the heterogeneity of TBI pathophysiology (43, 45), our study advances this framework by proposing specific, quantifiable threshold values derived from the integration of our cohort-derived IL-6 data and clinically validated GFAP reference standards. This refinement moves beyond broad theoretical associations to define a discrete, testable high-risk phenotype, offering a preliminary actionable reference for emergency risk stratification of TBI.

From a therapeutic perspective, the IL-6 trans-signaling pathway is a key driver of neuroinflammation exacerbation and BBB dysfunction (46). In mouse models of TBI, systemic administration of sgp130-Fc, a selective IL-6 trans-signaling inhibitor, improved learning and memory, alleviated anxiety-like behaviors, and reduced the levels of inflammatory chemokines in the brain parenchyma (8). Clinical studies in neuromyelitis optica spectrum disorder (NMOSD) have shown that satralizumab, an anti-IL-6 receptor monoclonal antibody, can cross the impaired BBB, inhibit T cell migration, and restore barrier function (47). Olamkicept, another highly selective IL-6 trans-signaling inhibitor, has shown promising clinical translation potential in inflammatory bowel disease (48), and its application in acute brain injury warrants further exploration.

For infectious and inflammatory conditions, C-reactive protein (CRP) and PCT are the standard clinical biomarkers for systemic bacterial infection and sepsis. Compared with CRP, IL-6 rises earlier in the inflammatory cascade (peaking at 1–2 hours after inflammatory stimulation vs. 6–8 hours for CRP) and correlates more strongly with the severity of CNS infections. However, PCT has superior specificity to IL-6 for differentiating bacterial from viral infections (49, 50). It must be emphasized that IL-6 alone cannot reliably differentiate infectious from vascular neurological disorders, as we observed substantial overlap in median IL-6 levels between the ischemic stroke group (14.00 pg/mL) and the CNS infection group (8.34 pg/mL) in our cohort. Accurate diagnosis therefore requires the integration of PCT, CRP, and clinical signs of infection.

Based on our findings, we further propose a hypothesis-generating clinical decision framework for serum IL-6 level interpretation (requires prospective multi-center validation) in **Table 6**, which provides a preliminary reference for frontline clinicians.

**Table 6:** Proposed hypothesis-generating clinical decision framework for serum IL-6 interpretation (requires prospective validation).

IL-6 level stratification	Clinical interpretation recommendations	Recommended follow-up actions	Evidence level
< 4 pg/mL	Extremely low probability of acute brain injury; may be seen in chronic conditions such as neurodegenerative diseases	Interpret in clinical context; consider CSF inflammatory marker testing if chronic neurodegenerative disease is strongly suspected	Retrospective cohort data; requires prospective validation
4–10 pg/mL	Mild systemic inflammation; requires vigilance for CNS infection or ischemic cerebrovascular events	Perform comprehensive infection screening; conduct cranial CT/MRI if clinically indicated	Retrospective cohort data; requires prospective validation
10–20 pg/mL	Strong signal of acute brain injury (TBI/hemorrhagic stroke)	Immediately perform cranial CT imaging and neurological evaluation	Retrospective cohort data; requires prospective validation
> 20 pg/mL	Highly suggestive of TBI, metabolic/toxic encephalopathy, or hemorrhagic stroke (highest probability for TBI, median 59.93 pg/mL)	Emergency neuroimaging evaluation and urgent neurology consultation	Retrospective cohort data; requires prospective validation

### 4.2 Study limitations

Several limitations of this study must be acknowledged:

1. Spectrum bias: The high diagnostic performance of IL-6 for distinguishing acute brain injury from healthy controls may not translate directly to unselected emergency department populations, where discrimination against other acutely ill patients (e.g., sepsis, myocardial infarction, polytrauma, post-surgical states) with elevated IL-6 levels is required. The limited discriminatory power of IL-6 for differentiating TBI from other neurological diseases (AUC = 0.741) further indicates that IL-6 cannot be used as a standalone triage tool in real-world emergency settings. Prospective validation in real-world emergency cohorts is necessary to confirm the clinical utility of IL-6 as a preliminary screening tool.
2. Residual confounding from systemic inflammation and comorbidities: As IL-6 is a pan-inflammatory cytokine, we cannot fully exclude the influence of concurrent systemic inflammation (e.g., post-injury infection, pneumonia, urinary tract infection) on serum IL-6 levels in the neurological disease groups. Due to the retrospective design, complete systematic comorbidity data were not collected for all participants, and we were unable to perform sensitivity analysis further excluding patients with concurrent in-hospital systemic infection, extracranial polytrauma, or sepsis-associated encephalopathy, which were not fully documented in the retrospective electronic health records. Future prospective studies with comprehensive comorbidity documentation are warranted to validate the disease-specific neuroinflammatory signature of IL-6.
3. Heterogeneity in sampling timing: IL-6 exhibits hours-scale kinetic changes after acute injury, and our study only included a single measurement within 24 hours of admission, with no systematic documentation of the exact time from injury to sampling for the TBI cohort. We were therefore unable to evaluate the effect of sampling timing on IL-6 levels and diagnostic performance. Future prospective studies with serial IL-6 measurements at predefined time points are needed to characterize its dynamic profile.
4. Generalizability limitation: This single-center study was conducted in the Han Chinese population, which may limit the generalizability of our findings across different ethnic groups and healthcare settings. Multicenter, multi-ethnic prospective cohorts are needed to validate our results and establish universal age- and sex-stratified reference intervals.
5. Lack of head-to-head biomarker comparison: We did not perform simultaneous detection of other established neurobiomarkers (GFAP, CRP, PCT), and thus could not evaluate the incremental diagnostic value of IL-6 beyond these routine clinical markers. This will be addressed in our future prospective studies.
6. Potential selection bias: The “other cerebrovascular diseases” and “other neurological disorders” groups were excluded from primary analysis due to ambiguous classification and small sample size, which may introduce a degree of selection bias. Future studies with refined disease classification are needed to explore IL-6 expression profiles in these rare neurological disorders.

## 5 Conclusion

In this large-scale cohort study of 6,465 individuals, we systematically characterized serum IL-6 expression profiles across 10 neurological disease categories using a single uniform detection platform. We demonstrate that serum IL-6 is a neuroinflammatory biomarker with robust efficacy for screening acute brain injury from healthy status. Age (U-shaped association) and sex are independent predictors of IL-6 levels, and their incorporation significantly improves diagnostic performance for CNS infections. Serum IL-6 holds potential as a preliminary screening indicator for acute neurological conditions in specific clinical settings, with clinical interpretation requiring age- and sex-stratified reference intervals. The diagnostic utility of IL-6 in unselected emergency triage settings, as well as the proposed clinical decision framework, require prospective validation in multi-center, real-world cohorts.

## Author's note

This study is a retrospective observational non-interventional cohort study, which does not meet the World Health Organization (WHO) definition of a clinical trial, so clinical trial registration is not required.

## Data Availability

The original contributions presented in the study are included in the article/**Supplementary Material**. Further inquiries can be directed to the corresponding author.

## Supplementary material

Supplementary Table 1 Serum IL-6 levels across neurological diseases stratified by sex and age subgroups.

Supplementary Table 2 Diagnostic performance of serum IL-6 for pairwise differential diagnosis between neurological diseases.

Supplementary Table 3 Subgroup analysis of serum IL-6 diagnostic performance for differential diagnosis between neurological diseases.
